# Unveiling the power of microenvironment in liver regeneration: an in-depth overview

**DOI:** 10.3389/fgene.2023.1332190

**Published:** 2023-12-13

**Authors:** Yuelei Hu, Ruilin Wang, Ni An, Chen Li, Qi Wang, Yannan Cao, Chao Li, Juan Liu, Yunfang Wang

**Affiliations:** ^1^ Department of Hepatobiliary and Pancreatic Surgery, The First Hospital of Jilin University, Jilin University, Changchun, China; ^2^ Hepato-Pancreato-Biliary Center, Beijing Tsinghua Changgung Hospital, School of Clinical Medicine, Tsinghua University, Beijing, China; ^3^ Department of Cadre’s Wards Ultrasound Diagnostics, Ultrasound Diagnostic Center, The First Hospital of Jilin University, Jilin University, Changchun, China; ^4^ Clinical Translational Science Center, Beijing Tsinghua Changgung Hospital, Tsinghua University, Beijing, China; ^5^ College of Life Science and Bioengineering, Faculty of Environmental and Life Sciences, Beijing University of Technology, Beijing, China

**Keywords:** hepatic lobule, regenerative microenvironment, liver regeneration, zonation, genes

## Abstract

The liver serves as a vital regulatory hub for various physiological processes, including sugar, protein, and fat metabolism, coagulation regulation, immune system maintenance, hormone inactivation, urea metabolism, and water-electrolyte acid-base balance control. These functions rely on coordinated communication among different liver cell types, particularly within the liver’s fundamental hepatic lobular structure. In the early stages of liver development, diverse liver cells differentiate from stem cells in a carefully orchestrated manner. Despite its susceptibility to damage, the liver possesses a remarkable regenerative capacity, with the hepatic lobule serving as a secure environment for cell division and proliferation during liver regeneration. This regenerative process depends on a complex microenvironment, involving liver resident cells, circulating cells, secreted cytokines, extracellular matrix, and biological forces. While hepatocytes proliferate under varying injury conditions, their sources may vary. It is well-established that hepatocytes with regenerative potential are distributed throughout the hepatic lobules. However, a comprehensive spatiotemporal model of liver regeneration remains elusive, despite recent advancements in genomics, lineage tracing, and microscopic imaging. This review summarizes the spatial distribution of cell gene expression within the regenerative microenvironment and its impact on liver regeneration patterns. It offers valuable insights into understanding the complex process of liver regeneration.

## 1 Introduction

Organisms frequently encounter tissue damage and possess remarkable regenerative capabilities. Tissue repair post-injury can lead to the complete restoration of structure and function through similar cell types or result in the formation of fibrous tissue and scarring. Stem cells often play a pivotal role in this complex process of tissue repair (Dekoninck and Blanpain, 2019). However, the liver stands out as a unique organ with a tissue repair mechanism distinct from that of other organs. The liver performs complex physiological and biochemical functions and can be categorized into hepatic parenchymal cells and non-parenchymal cells. Non-parenchymal cells create a supportive microenvironment for the health and function of parenchymal cells. The liver is considered an ‘injury-privileged’ organ, as it can rapidly undergo liver regeneration (LR) and restore its function following surgical resection or drug-induced hepatocyte loss (Gao and Peng, 2021).

LR is an extremely complex biological process involving the proliferation and hypertrophy of hepatocytes and the proliferation of non-parenchymal cells in the liver. This intricate process relies on a complex network of inflammatory and growth factors (Michalopoulos and Bhushan, 2021). Hepatic lobules exhibit near-perfect anatomical structures and functional segmentation, serving as the foundation for liver function. When viewed in two dimensions, they form a hexagonal structure created by the connection of six portal areas surrounding the central vein. Hepatic lobules are primarily divided into three zones, with Zone 1 hepatocytes near portal vein (PV) areas and connecting points, Zone 3 hepatocytes around the central vein (CV), and Zone 2 hepatocytes positioned between them (Ben-Moshe and Itzkovitz, 2019; Paris and Henderson, 2022). Numerous studies have highlighted the central role of mature hepatocytes in the LR process. Liver progenitor cells can also significantly contribute to LR. Some subpopulations of liver stem cells with Lgr5, further add to the liver’s regenerative process (Ang et al., 2019). The expression of embryonic gene clusters suggests that the regenerative process involves a multitude of repetitive molecular network interactions akin to liver development and growth (Chen et al., 2020; Chembazhi et al., 2021; Ben-Moshe et al., 2022).

It is important to recognize that LR and development and growth are distinct biological processes. By employing lineage tracing techniques, researchers have found that hepatocytes in Zone 2 are the primary source of hepatocytes responsible for maintaining liver homeostasis under normal conditions. In cases of regional liver injury, LR is supplemented by the proliferation of hepatocytes in the non-injured area (Wei et al., 2021). Recent research into LR induced by acetaminophen (APAP) has shown that early proliferating hepatocytes tend to concentrate in the PV region. By the 72-h mark, proliferation levels in all three areas equalize, suggesting that substantial hepatocyte proliferation along the lobular axis exerts mitotic pressure, facilitating rapid hepatocyte relocation to the injured area and achieving the goal of cell replacement (Ben-Moshe et al., 2022). Likewise, a preliminary investigation into LR following partial hepatectomy in mice revealed that early proliferating hepatocytes primarily concentrate in Zone 2, with additional proliferating hepatocytes in Zones 1 and 3 (Chembazhi et al., 2021).

Distinct patterns of LR emerge in response to different types of liver injury. In instances of regional liver injury, substantial liver tissue loss is absent, and damage is primarily limited to regional hepatocyte necrosis within the hepatic lobule. Conversely, non-regional liver injuries, such as partial hepatectomy, do not involve the loss of regional hepatocytes. These differences in the nature of liver injury result in distinct LR patterns. In 1985, an innovative ‘streaming liver’ model was proposed by some scholars but was quickly rejected (Zajicek et al., 1985; Bralet et al., 1994). Interestingly, when non-regional liver injuries are comprehensively considered in both temporal and spatial dimensions, they exhibit similarities to the “streaming liver” model. However, this is mainly characterized by the rapid dispersion of proliferating hepatocytes rather than the flow of hepatocytes. Essentially, it represents a time-series change in cells driven by differences in mobility and signal molecule expression within the liver regeneration microenvironment. In this review, we provide an overview of the spatial differences in gene expression within liver lobules. Additionally, we classify and describe injury models based on their outcomes, laying the foundation for a deeper understanding of LR. We summarize the varying contributions of hepatocytes and liver progenitor cells to LR in spatial dimensions. In the temporal dimension, we explore the evolution of the LR process. Furthermore, we delve into the cell interaction dimension, introducing the signaling molecules and pathways that regulate LR in the regenerative microenvironment. This review aims to consolidate recent progress in understanding liver regeneration models and provide valuable insights for a deeper understanding of this intricate process.

## 2 Liver development, growth, and regeneration

The formation of gastrula led to the division of embryonic cells into three germ layers, ectoderm, mesoderm and endoderm. The lumen formed around the endoderm is the original intestinal lumen, and the endoderm is the main cell source for the development of the internal organs (Wells and Melton, 1999). The mesoderm-derived signaling molecules, such as BMP, Wnt, FGF and retinol, are involved in the development of liver buds at the terminal foregut (Jung et al., 1999; Wang et al., 2015; Palaria et al., 2018). Matrix metalloproteinases (MMP) such as MMP2 and MMP14 hydrolyze the basement membrane around the endoderm. Proliferating hepatoblasts maintain cell-to-cell junctions and migrate in a cord-like manner, and mix with endothelial cells and mesenchymal cells in the process. The subsequent organogenesis of the liver produced complex structures, including extensive differentiation of parenchymal and non-parenchymal cell types, development of the biliary tract, sinus capillaries, and vascular system, and tissue of the extracellular matrix. The detailed process of liver development has been summarized by Lotto et al. (2023). In mice, the structure and function of hepatic lobules are gradually improved after birth. Hepatocytes are still in the stage of rapid proliferation within 1 week after birth. High expression of embryonic genes such as *Afp, Ahsg* and *H19* and cell proliferation genes such as *Mki67, Mybl2, Top2a* and *Ccnd1* indicates that hepatocytes during this period are still not mature hepatocytes. However, at 21 days, although the proliferation of hepatocytes and the expression of hepatoblast marker genes were downregulated, the expression of mature hepatocyte genes such as *Hnf4a, Cyp1a2*, and *Cyp3a11* was still low, and the hepatocytes were in a transitional state. According to the expression of *Cyp2e1, Cyp2f2, Glul* and *Cdh1*, the functional zone of hepatocytes in the hepatic lobule is very clear at 56 days after birth, and it is not clear at the early time point (Liang et al., 2022). This shows that although the cell arrangement and tissue structure in the hepatic lobule are perfect, its function is not yet mature, and this clear functional distribution is gradually improved after birth. However, for rats, the liver weight continued to increase within 30 days after birth, but the liver/body weight ratio decreased first within 10 days and gradually increased after 10 days (OLIVER et al., 1962). We stained the liver tissue of rats within 6 weeks after birth and found that Ki67 positive hepatocytes were dispersed in the liver tissue, but CyclinD1 positive hepatocytes showed more hepatocytes in Zone 1 and Zone 2 and less hepatocytes in Zone 3 within 2 weeks. In the growth state, Ki67 and CyclinD1 labeled two different distribution states of proliferating cells. After being damaged by biological (viruses, parasites, *etc.*) and non-biological (toxins, drugs, PH), the liver has a strong ability to regenerate. Recent studies have shown that fetal gene expression is induced in both regional liver injury caused by APAP and non-regional liver injury caused by PH, suggesting that the process of LR has some similar biological processes with the development and growth stages of the liver (Chembazhi et al., 2021; Ben-Moshe et al., 2022). LR is a complex biological process, which requires the participation of a variety of inflammatory factors and growth factors. The completion of LR can be completed by two processes: hepatocyte division and hypertrophy (Michalopoulos and Bhushan, 2021). The reasons for the complexity of the LR process are multifaceted, mainly involving the diversification of the source of regenerative hepatocytes, the complex LR microenvironment and the gastrointestinal flora, nutrient supply, vagus nerve, mechanical force changes, and information exchange with other organs (Xu et al., 2022; Hu et al., 2023).

## 3 Structure and zonation of liver lobules

While liver development and morphology vary across different species, the fundamental structural unit of the liver remains remarkably similar. The liver is composed of repetitive anatomical units known as liver lobules, each of which takes the form of a hexagonal column. In mice, these lobules have a diameter of approximately 0.5 mm, while in humans, the diameter is around 1 mm (Teutsch, 2005; Hoehme et al., 2010). Blood enters the liver lobules from the periportal area through both the PV and hepatic artery. It then proceeds through the hepatic sinusoids to drain into the central vein within each liver lobule. Ultimately, the blood exits the liver (Hoehme et al., 2010). However, the drainage direction of bile is opposite to that of blood. It primarily accumulates in the portal triads and exits the liver through the biliary drainage system into the digestive tract. The flow of fluids imparts directionality to the liver lobules within a two-dimensional plane. Blood flow delivers oxygen, nutrients, and cytokines, and its own fluidity and hydrostatic pressure also bring shear stress and tension. Physical and biological factors in space bring differential distribution to the above physical and non-physical indicators, such as oxygen consumption, hormone inactivation, blood flow rate and pressure reduction. In terms of oxygen consumption, the oxygen tension around the PV decreased from 65 to 30 mmHg in the pericentral layer (Kietzmann et al., 2006). These changes produce spatial gradient differences in hepatic lobules, and affect the changes in gene expression profiles of hepatocytes, endothelial cells, *etc.* The main cell types in the liver are hepatocytes, liver endothelial cells, hepatic stellate cells (HSCs), and Kupffer cells. Among these, hepatocytes, liver endothelial cells, and HSCs are the primary focus of zonal distribution studies.

### 3.1 Zonal distribution of hepatocytes

**FIGURE 1:**
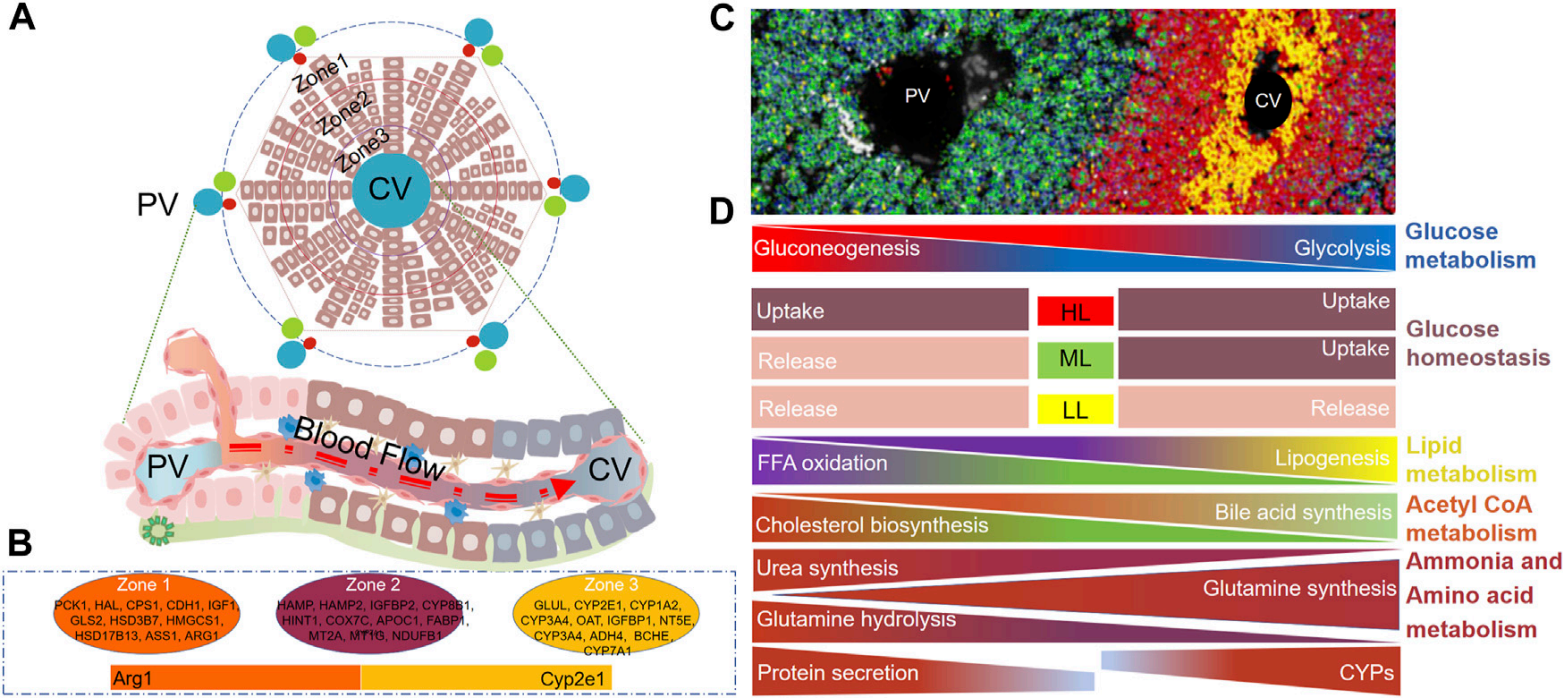
Structure of Liver lobules and zonation of hepatocyte genes and functions. **(A)** Two-dimensional schematic representation of liver lobule structure, from outer to inner regions, denoted as Zone 1, Zone 2, and Zone 3. **(B)** Zonation of marker gene in hepatocytes within Zone 1, Zone 2, and Zone 3 of the liver lobule. **(C)** PV - CV image in mouse liver tissue, Reprinted with permission from Hu et al. (2022), licensed under CC BY 4.0. **(D)** Functional zonation of hepatocytes within liver lobules. PV, Portal vein, CV, Central vein, HL, High level, ML, Medium level, LL, Low level.

Hepatocytes are the parenchymal cells of the liver, accounting for 80% of the liver weight. In terms of the number of cells, they account for about 60% of the total number of liver cells (Godoy et al., 2013). In mice, about half of the genes in hepatocytes are expressed regionally (3,500 of the 7,000 genes are differentially expressed along the lobules), and most of them show a gradient change (Inverso et al., 2021). The application of advanced technologies aids in the identification of markers for hepatocytess in different zones of the liver lobules, leading to a significant advancement in our understanding of liver lobules. At the gene expression level, several markers are highly expressed in distinct regions of the liver lobules. In the PV area hepatocytes, genes such as *Pck1, Hal, Cps1, Cdh1, Igf1, Gls2, Hsd3b7, Hmgcs1, Hsd17b13, Ass1, Arg1, G6pc, Glut2, Sds, Cyp2f2, Sox9,* and *Alb* exhibit high expression. In CV area hepatocytes, high expression is observed for genes including *Cyp7a1, Glul, Cyp2e1, Cyp1a2, Cyp3a4, Oat, Igfbp1, Nt5e, Adh4, Bche, Gck, Slc1a2, Cyp2a5,* and *Glut1*. Moreover, genes such as *Hamp, Hamp2, Igfbp2, Cyp8b1, Hint1, Cox7c, Apoc1, Fabp1, Mt2a, Mt1g*, and *Ndufb1* display high expression in mid-lobule hepatocytes (Poliard et al., 1986; Jungermann and Katz, 1989; Ben-Moshe and Itzkovitz, 2019; Hildebrandt et al., 2021; Paris and Henderson, 2022; Hu et al., 2023; Martini et al., 2023). The differentiation of hepatocytes within distinct zones of the liver lobules maybe a result of the interplay of four key factors: the physiological direction of blood flow, the mutual exclusivity of functional roles, differential gene expression, and the influence of the local microenvironment. This spatially distinct gene expression pattern within the liver lobules assigns specific functions to various hepatocytes, facilitating a cooperative and complementary approach that maximizes the liver’s overall functionality. In terms of glucose metabolism, hepatocytes in the PV region are responsible for gluconeogenesis (*Pkc, G6pc* gene expression), and hepatocytes in the CV region are mainly responsible for glycolysis (*Gck* gene expression) (Katz et al., 1977; Bahar Halpern et al., 2015). In the context of glucose homeostasis, elevated blood glucose levels prompt glucose absorption in all hepatocytes. Conversely, low blood glucose level trigger glucose release from all hepatocytes. Interestingly, under moderate blood glucose levels, hepatocytes in the PV area release glucose, while those in the CV area absorb it (Jungermann et al., 1982; Jungermann and Katz, 1989; Berndt et al., 2018). In the context of lipid metabolism, hepatocytes in the PV area are primarily responsible for the oxidative metabolism of fatty acids, while hepatocytes in the CV area are predominantly engaged in lipid synthesis (Katz et al., 1983). It is intriguing to note that evidence suggests that hepatocytes in the PV area exhibit high expression of L-FABP, a key player in fatty acid transport, which likely contributes to the oxidative metabolism of fatty acids in the PV area (Suzuki and Ono, 1987; Bass et al., 1989). The primary substrate for cholesterol synthesis is acetyl-CoA, which coincidentally serves as an intermediate in fatty acid beta-oxidation metabolism. Moreover, the relatively high expression of hydroxymethylglutaryl-CoA (HMG-CoA) reductase in hepatocytes from the PV area supports the notion that cholesterol is synthesized by hepatocytes in PV area, given that acetyl-CoA is a fundamental substrate for this process. Conversely, the expression of *Cyp7a1* in the CV area serves as a pivotal enzyme in cholesterol metabolism, leading to the predominant synthesis of bile acids in CV area hepatocytes. This highlights the role of CV area hepatocytes in the conversion of cholesterol into bile acids (Kang and Davis, 2000). ammonia is a toxic byproduct produced during the body’s protein metabolism. The liver plays a vital role in metabolizing ammonia into a non-toxic substance that can be subsequently excreted from the body. Hepatocytes located in the PV area primarily facilitate the conversion of ammonia into urea, thus contributing to its detoxification (Pösö et al., 1986). Glutamine is an amino acid in mammals, and its metabolic is a dynamic process in the liver. Hepatocytes in the PV region decompose glutamine to produce partial ammonia (*Gls2* gene expression advantage). Hepatocytes in the CV region mainly synthesize glutamine (*Glul* gene expression advantage) (Hakvoort et al., 2017; Paluschinski et al., 2021). It is important to note that the synthesis of glutamine is also a significant detoxification pathway for ammonia to some extent. Research indicates that approximately 35% of ammonia is detoxified through the synthesis of glutamine, while another 35% is detoxified through urea synthesis (Hakvoort et al., 2017). The liver is the main place for plasma protein synthesis in the body, mainly in the PV region (Poliard et al., 1986; Ben-Moshe and Itzkovitz, 2019; Payen et al., 2021). Interestingly, some studies have found that the hepatocytes in the PV area are also mainly related to iron homeostasis (Payen et al., 2021). Another important role of the liver is detoxification. The CV region expresses a large amount of *Cyp2e1*, so it undertakes the main drug metabolism function, which is also the main reason for the liver injury caused by carbon tetrachloride (CCL4) and APAP mainly around the CV region (Diaz Gómez et al., 2006; Hu et al., 2022). The structure of liver lobules and the zonation of gene expression and functions in hepatocytes are illustrated in Figure 1.

### 3.2 Zonal distribution of endothelial cells

**FIGURE 2:**
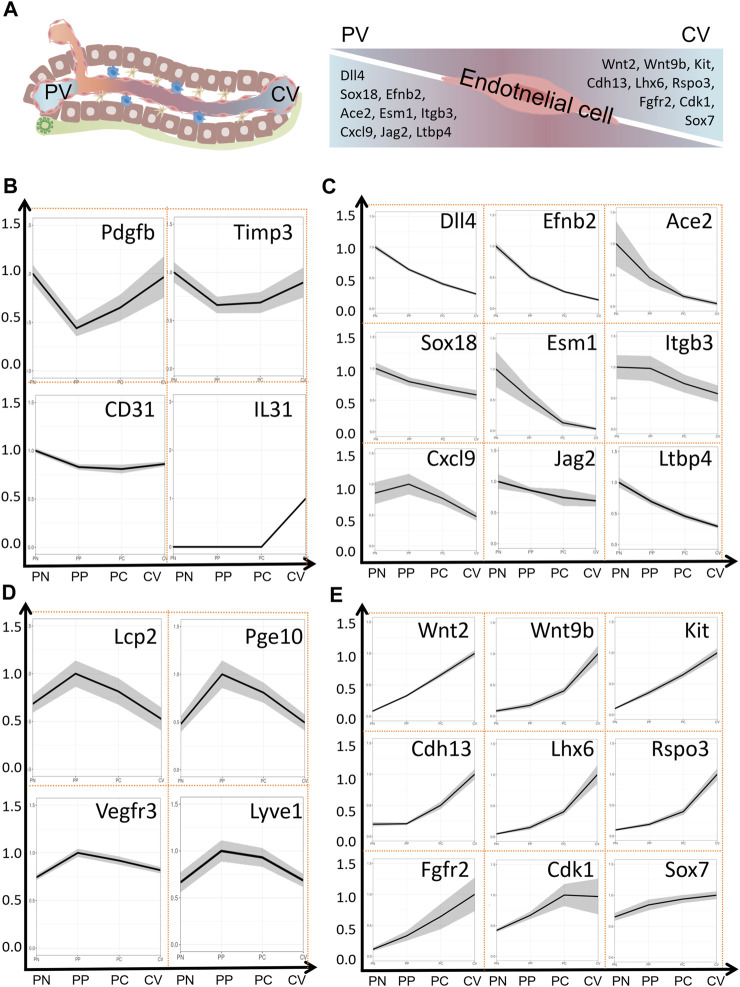
Zonation of Gene Expression in Endothelial Cells of Liver Lobules. **(A)** Liver lobule structure and expression of marker genes in the PV area and CV area within endothelial cells. **(B)** Expression of marker genes in major blood vessels within the liver. **(C)** Expression of marker genes in the PV area. **(D)** Expression of marker genes in blood sinusoidal endothelial cells. **(E)** Expression of marker genes in the CV area. Data sourced from a publicly accessible online database at https://pproteomedb.dkfz.de created by Inverso et al. (2021). PN, portal node, PP, peri-portal, PC, peri-central, CV, central vein, PV, portal vein, CV, central vein.

In the liver, apart from the regional distribution of hepatocytes, liver endothelial cells also exhibit significant zonal distribution. In fact, the zonation of endothelial cells is considered to be one of the initiating factors for the zonation of hepatocytes (Rocha et al., 2015; Ko and Monga, 2019; Inverso et al., 2021). Hepatic endothelial cells make up around 20% of the liver cell population, constituting approximately 50% of non-parenchymal cells (Aird, 2007). They form the liver’s vascular network, clear toxins and bacteria, regulate immune responses, present antigens, secrete cytokines, and influence hepatocyte function (Aird, 2007; Rafii et al., 2016; Strauss et al., 2017). Hepatic endothelial cells consist mainly of vascular endothelial cell and liver sinusoid endothelial cells (LSEC), with a small presence of lymphatic endothelial cells. LSEC form the sinusoidal wall. Unlike typical capillaries, LSEC lack an organized basement membrane, rendering liver microvascular endothelium discontinuous. However, in chronic liver diseases, LSEC may lose their unique fenestration features, becoming capillarized, thus disrupting their role in maintaining the quiescent state of HSCs and contributing to liver fibrosis (Poisson et al., 2017). Proteomic analysis reveals that the expression of cell adhesion molecules and tight junction proteins is primarily in large vascular endothelium. These findings suggest that LSEC represent a distinct cell population compared to the large vascular endothelium. For instance, *Peg10, Lcp2, Flt-4 (VEGFR3)*, and *Lyve1* exhibit higher expression in LSECs, while *PECAM1 (CD31)*, *IL-33*, *Pdgfb*, and *Timp3* show relatively higher expression in large vascular endothelium (Inverso et al., 2021). Research indicates that gene expression in LSECs is not uniformly distributed along the liver lobules. Approximately 40% (4,943/13,737 or 475/1,300) of genes are expressed in a zonated manner (Halpern et al., 2018; Inverso et al., 2021). According to the location of blood vessels and the direction of blood flow drainage, hepatic endothelial cells can be divided into four subgroups as follows: portal node (PN), peri-portal (PP), peri-central (PC), central vein (CV). *Sdc1, Esm1, Ace2, Angpt2, Cxcl9* were highly expressed in endothelial cells of PN region, and *Lhx6, Wnt2, Fgfr2,* and *Cdk1* were highly expressed in endothelial cells of CV region. It is noteworthy that in endothelial cells, the expression of many genes gradually changes along the spatial structure of the liver lobules. Genes such as *Lhx6, Wnt2, Fgfr2, Sox7, Kit (CD117), Wnt9b, Rspo3, Cdh13,* and *Cdk1* display higher expression levels as they progress spatially toward the CV. In contrast, genes like *Sox18, Esm1, Ace2, Cxcl9, Dll4, Efnb2*, and *Itgb3* show decreasing expression levels as they progress toward the CV area. Proteomics results suggest that about 25% of the protein expression is regionally expressed. Interestingly, the metabolic enzymes of the CYP family are mainly distributed in the PV region, which is not consistent with the main distribution of hepatocytes in the CV region. Phosphorylation predominantly occurred on serine (S), threonine (T), and tyrosine (Y) residues (Sharma et al., 2014). These residues accounted for 77%, 20%, and 3% of the identified phosphorylation sites, respectively. Phosphoproteomic analysis revealed that phospho-serine (p-S) and phospho-threonine (p-T) showed no differential expression across liver lobules, whereas the expression of 117 phospho-tyrosine residues exhibited a clear zonal pattern, with TIE1 being a representative example (Inverso et al., 2021). The zonation of gene expression in endothelial cells and the highly expressed genes in different regions of endothelial cells are depicted in Figure 2.

### 3.3 Zonation distribution of HSCs

**FIGURE 3:**
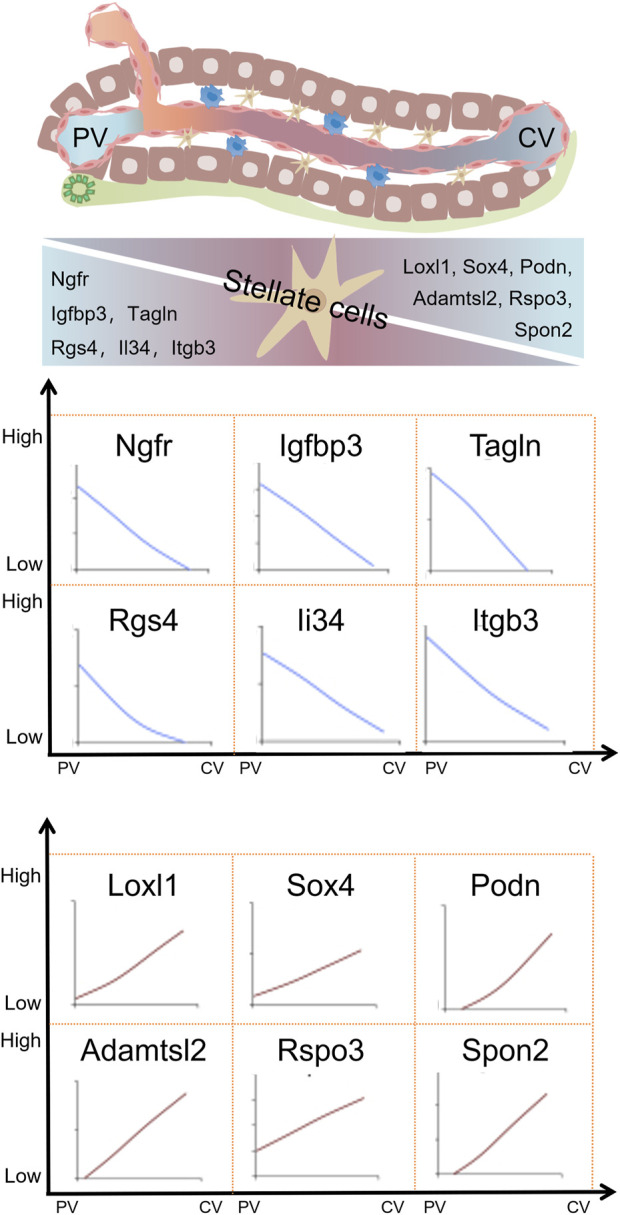
Zonation of gene expression in hepatic stellate cells within liver lobules. Hepatic stellate cells in liver lobules also exhibit zonal gene expression, with variations in gene expression levels demonstrating distinct expression patterns around the portal vein (PV) and central vein (CV) areas. Adapted with permission from Dobie et al. (2019), licensed under CC BY 4.0.

The accuracy of identifying HSCs is confirmed through the specific expression of established stellate cell marker genes such as *Rgs5, Ptn, Ngfr, Lrat, Fbln5, Dpt, Dcn, Cygb, and Colec11*. Further classification of HSCs based on differences in gene expression revealed that HSC had two different subgroups. HSC1 showed highly expression of cell surface proteoglycan *Gpc3*, the neurotrophic receptor *Ntrk2, Efemp1, Gem, Ccl2*, and *Thbs1*. HSC2 exhibited elevated expression of several key genes, including the dopamine norepinephrine-converting enzyme *Dbh*, the hedgehog signaling modulator *Hhip*, as well as G-protein-coupled receptors that are targeted by vasorelaxation peptide hormones, *Vipr1, Pth1r, Ramp1, Ednrb,* and *Agtr1a*. In addition to the above differential gene expression, HSC1 and HSC2 expressed different levels of key secreted cytokines (*Ccl2, Ccl21* and *Il32*), chemokines (*Cxcl12* and *Cxcl14*), angiogenins (*Angptl1, Angptl2* and *Angptl6*) and mitogens (*Hgf, Hdgf, Vegfc and Pgf*) (Payen et al., 2021). These evidences indicate the heterogeneity of gene expression in HSCs in the liver. Further studies have shown that the gene expression of HSCs is also expressed in a zonal distribution. *Ngfr, Tagln, Igfbp3, Rgs4, Itgb3* and *Il34* were mainly expressed in HSCs in PV area, while *Adamtsc2, Rspo3, Spo2, Podn, Sox4* and *Loxl1* were mainly expressed in CV area (Dobie et al., 2019). The similarity of *Itgb3* and *Rspo3* expression in hepatic vascular endothelial cells and HSCs also indicates that there is a gene expression profile determined by certain environmental factors (Dobie et al., 2019; Inverso et al., 2021). Secondly, it is well known that endothelial cells can also inhibit the activation of HSCs in the body (Maretti-Mira et al., 2019). The space heterogeneity of gene expression profiles of various cells in the liver is the basis for the complex function of the liver. The zonation of gene expression in hepatic stellate cells within the liver is presented in Figure 3.

### 3.4 Zonal distribution of Kuppfer cells

Kupffer cells (KC) are resident macrophages in the liver, playing a crucial role in the innate immune system of the liver. KC are primarily distributed in the space between the LSECs and hepatocytes, where they come into contact with antigens from the digestive tract. In rats, the distribution of KC in the liver sinusoids is not uniform. Approximately 43% are distributed in the PV zone, 28% in the midzone, and 29% in the CV zone (Bouwens et al., 1986). It is interesting to note that studies in both mice and humans also indicate that KC predominantly distribute around the PV zone within the liver lobule (MacParland et al., 2018; Gola et al., 2021). In specific pathogen-free (SPF) mice, there is a clear presence of immune zonation, while in germ-free mice, there is no apparent phenomenon of immune zonation. However, when germ-free mice are co-housed with SPF mice, there is a significant induction of immune zonation. Similarly, administering antibodies to SPF mice leads to the disappearance of immune zonation. Further research reveals that the zoning of KC within the liver lobule is dynamic and primarily influenced by substances from the digestive tract, such as lipopolysaccharides. During the process of immune zonation, endothelial cells play a crucial role as the key mediator cells in its formation (Gola et al., 2021). However, recent research utilizing spatial transcriptomics techniques suggests that the distribution of KC within the liver lobule is not strictly regional (Hildebrandt et al., 2021). Currently, there is limited research on the spatial differences in gene expression related to the distribution of KC within the liver lobule. Therefore, the spatial characteristics of KC in the liver lobule still require further investigation and clarification.

## 4 Injury models for LR

Various animal models and liver injury methods have been employed to study LR, resulting in differing regenerative effects due to the differences in animal models and injury mechanisms. Here, we summarize the models used to induce LR. Liver injury methods can be broadly divided into two categories: surgical-induced injury, which includes procedures like PH, portal vein ligation (PVL), Associating Liver Partition and Portal Vein Ligation for Staged Hepatectomy (ALPPS), partial liver transplantation, and portal vein embolization (PVE). The other category involves non-surgical injury, primarily caused by drug-induced hepatocyte death from substances such as alcohol allyl (AA), acetaminophen (APAP), CCl4, and 3,5-diethoxycarbonyl-1,4-dihydro-collidine (DDC). Various animal models have been utilized for studying LR, including zebrafish, mice, rats, rabbits, and pigs (Liao et al., 2017; Daradics et al., 2021; Jung et al., 2021; Oldhafer et al., 2021; Zhang et al., 2021). Liver injury induced by drugs and toxins is primarily due to hepatocyte necrosis. For instance, CCL4 and APAP mainly cause injury to CV hepatocytes, while DDC and AA primarily induce injury to PV hepatocytes (Reid, 1972; Zhu et al., 2013; Wei et al., 2021; Ben-Moshe et al., 2022). Regional liver injury-induced LR is primarily mediated by the proliferation of hepatocytes in non-injured areas. Surgical liver injury can be divided into two categories, one of which lead to acute loss of hepatocytes, such as PH and partial liver transplantation. The other category involves non-acute loss of hepatocytes, such as PVE, ALPPS, PVL, and so on. Surgical-induced LR is induced by the re-entry of existing cells in the residual liver into the cell cycle for proliferation. The research model of LR is summarized in Table 1.

**TABLE 1:** The similarities and differences of different liver regeneration models.

Category	Injury mode	Hepatocyte loss	Range	Injury mechanism	Liver lobule increase/hypertrophy	Acute changes in the blood flow
Chemical induce	AA (Reid, 1972)	Hepatocytes necrosis	PV Zone	Covalent binding of metabolites with liver cells in the PV region	No	No
	DDC (Li et al., 2023)			Fibrosis in the PV region		
	ANIT (Mariotti et al., 2018)	Chronic loss of hepatocytes		Fibrosis in the PV region		
	CCL4 (Diaz Gómez et al., 2006)	Hepatocytes necrosis	CV Zone	Metabolized by Cyp2e1 to produce reactive trichloromethyl radials		
	APAP (Chao et al., 2018)			Metabolized into NAPQI through Cyp2e1 and Cyp1a2 to consume gsh		
	TAA (Oliver et al., 2006)			Production of reactive oxygen species through Cyp2e1 metabolism		
Surgical induction	BDL (Zhang et al., 2017)	Chronic loss of hepatocytes	PV Zone	Fibrosis in the PV region	No	No
	PH (Langiewicz et al., 2017)	Acute loss of hepatocytes	Overall loss of the liver lobules	Resection of the liver tissue	Yes	Yes
	PLT (Liu et al., 2012)					
	PVL/PVE (Tranchart et al., 2016; Langiewicz et al., 2017)	Chronic loss of hepatocytes		Atrophy of the liver tissue		
	ALPPS (Langiewicz et al., 2017)					

AA, alcohol allyl, DDC, 3,5-diethoxycarbonyl-1, 4-dihydro-collidine, CCL4, carbon tetrachloride, ANIT, alpha-naphthylisothiocyanate, APAP, acetaminophen, TAA, thioacetamide, BDL, bile duct ligation, PH, partial hepatectomy, PLT, partial liver transplantation, PVL, portal vein ligation, PVE, portal vein embolization, ALPPS, associating liver partition and portal vein ligation for staged hepatectomy, PV, portal vein, CV, central vein, NAPQI, N-acetyl-p-benzoquinone imine.

### 4.1 Regional liver injury - CV zone liver injury model

CCl4 induced liver injury is usually carried out by intraperitoneal injection. It is one of the common models for studying acute and chronic liver injury (Chen et al., 2020; Wu et al., 2020). This liver injury model is widely used in hepatic pathology research, demonstrating morphological and biochemical features similar to cell lesions in human hepatic disease. The metabolism of CCl4 leads to the formation of highly reactive trichloromethyl radicals, which covalently bind to nuclear proteins, and Cyp2e1 is the primary enzyme responsible for metabolizing CCl4 (Diaz Gómez et al., 2006). Due to the zonated distribution of hepatocytes functions, Cyp2e1 are expressed at higher levels in hepatocytes around the CV. Consequently, a single dose of CCl4 can lead to necrosis of hepatocytes around the CV, while long-term administration can result in liver fibrosis, cirrhosis, and hepatocellular carcinoma (Chen et al., 2020; Wu et al., 2020).

APAP is one of the commonly used drugs with well-established hepatotoxicity (Budnitz et al., 2011; Herndon and Dankenbring, 2014). At conventional doses, APAP does not cause hepatocytes damage. However, when taken in excess, it can lead to severe liver injury (Clark et al., 2012). At therapeutic doses, the majority (about 90%) of APAP is primarily metabolized through phase II reactions (glucuronidation and sulfation) and excreted into the urine via the kidneys. The remaining approximately 10% is further metabolized by cytochrome P450 enzymes, primarily Cyp2e1 and Cyp1a2, to form the reactive metabolite N-acetyl-p-benzoquinone imine (NAPQI). Highly reactive NAPQI is rapidly conjugated with hepatic glutathione (GSH) and excreted into the bile, with no significant harmful effects on hepatocytes. However, in cases of APAP overdose, the glucuronidation and sulfation pathways become saturated, leading to an excess generation of NAPQI that depletes intracellular and mitochondrial GSH in the liver. The remaining NAPQI subsequently reacts with cellular macromolecules, particularly proteins, forming covalent bonds, resulting in mitochondrial damage and necrosis, ultimately leading to cell death (Chao et al., 2018).

### 4.2 Regional liver injury - PV zone liver injury model

The toxicity of AA results from its oxidation to acrolein by hepatic alcohol dehydrogenase (Serafini-Cessi, 1972; Rikans, 1987). Research has indicated that alcohol dehydrogenase is localized within hepatocytes surrounding the PV area of the liver lobule and is considered the primary reason for AA induced injury to PV zone hepatocytes (Reid, 1972). However, another study suggests that in trout liver, alcohol dehydrogenase is evenly distributed across all hepatocytes, with no significant variation (Schär et al., 1985). Some researchers also propose that the cause of AA induced PV zone hepatocellular injury is due to the covalent binding of AA metabolites to hepatocytes in the PV zone (Reid, 1972).

The liver injury model caused by DDC diet is mainly to simulate the manifestations of porphyria caused by abnormal porphyrin metabolism in human body. In rodents fed with DDC, interference in multiple steps of porphyrin metabolism results in the accumulation of porphyrins, intermittent biliary obstruction, cholangitis, and biliary fibrosis (Saggi et al., 2019). DDC primarily induces injury to the hepatocytes around the PV area, and both short-term (1 week) and long-term (6 weeks) feeding can be utilized in studies related to LR (Addante et al., 2018; Li et al., 2023).

### 4.3 Non-regional liver injury - Acute hepatocyte loss

PH is the main surgical treatment for malignant liver diseases (Alkhalili and Berber, 2014; Chan et al., 2020). After surgical removal of a portion of the liver, the acute loss of hepatic parenchymal cells disrupts the liver’s homeostasis, prompting the remaining cells in the residual liver to re-enter the cell cycle for proliferation in order to restore the liver’s weight and function (Kountouras et al., 2001). Rodents and zebrafish are commonly used to study LR caused by PH (Ypsilantis et al., 2020; Oderberg and Goessling, 2021; Wen et al., 2023), among which mice are widely used. According to the liver structure of mice, it can be divided into 7 liver lobes in detail, or simply divided into 4 lobes, namely, left lobe, middle lobe, right lobe and caudate lobe. Although 70% of PH models are the most commonly used models for studying LR, 30%, 50%, and 90% of PH are performed according to different experimental designs (Myronovych et al., 2008; Brandt et al., 2016; Shao et al., 2021). The literature shows that laparoscopic surgery has been carried out on this basis (Ypsilantis et al., 2020). It is interesting that laparoscopic PH has a higher promotion of mitotic activity during LR than open PH. While PH models in vertebrates still have limitations in extrapolating to human LR, they remain a classic and controlled model, thus serving as a significant tool in the field of LR research. Liver transplantation has emerged as a crucial treatment option for end-stage liver diseases. Advances in surgical techniques and immunosuppressive therapies have elevated liver transplant procedures to become a routine and effective treatment method (Meirelles Júnior et al., 2015; Di Maira et al., 2020). The shortage of available donor livers remains a significant limitation for liver transplant procedures, and it has substantial health implications for patients awaiting transplantation (Samuel and Coilly, 2018). Given the liver’s unique regenerative capacity, advancements in partial liver transplant techniques provide a favorable foundation for expanding liver transplant operation and increasing the donor pool. This, in turn, will enhance the treatment efficiency for end-stage liver diseases (Goldaracena and Barbas, 2019; Lerut, 2020). In animal experiments, partial liver transplantation is commonly employed to investigate the impact of different graft volumes on postoperative LR. This is because smaller graft volumes can often lead to small-for-size syndrome and insufficient regeneration (Wertheim et al., 2011; Liu et al., 2012).

### 4.4 Non-regional liver injury - Chronic hepatocyte loss

In patients, the performance of PH may be restricted when the remaining liver volume and function are insufficient (Garcea and Maddern, 2009; Chan et al., 2020). Hence, prior to surgery, techniques such as preoperative PVE and ALPPS are frequently employed to facilitate the regeneration of residual liver tissue (Yi et al., 2022). PVE is a procedure that uses embolic materials to block the blood vessels within the PV. The selection of embolic materials, whether they are temporary or permanent, can lead to different outcomes in terms of inducing LR (de Baere et al., 2009). PVE, PVL, and ALPPS can all induce varying degrees of LR. PVE with using permanent embolic materials and ALPPS procedures have shown promising outcomes in inducing LR in human cases (Vyas et al., 2014; Jaberi et al., 2016; Chan et al., 2021). PVE, PVL, and ALPPS share a common characteristic when inducing LR: they do not cause acute loss of hepatocytes, while a portion of the liver gradually undergoes atrophy due to the loss of PV blood supply. While several studies suggest that changes in PV blood flow are important factors in stimulating LR, it is interesting to note that there is evidence indicating that LR can still occur in the absence of PV blood flow (Wei et al., 2016).

## 5 Spatial dimension analysis of LR: Cell source

The maintenance of liver homeostasis primarily relies on the existing hepatocytes, with a very limited contribution from liver progenitor cells (Malato et al., 2011). While existing hepatocytes play a significant role in the process of LR following liver injury, researchs have revealed that in cases of severe liver damage and suppressed hepatocyte proliferation, biliary epithelial cells (BEC) can dedifferentiate to form liver progenitor cells with stem-like properties. These cells serve as a crucial source for mediating LR (Deng et al., 2018; Chen et al., 2020; Pu et al., 2023). During prolonged periods of chronic injury, BEC play a significant role in assisting LR by differentiating into hepatocytes through the formation of HNF4α^+^CK19^+^ bipotent cells. Furthermore, with the extended duration of the injury, this mechanism contributes more substantially to LR. However, when the injury ceases, the HNF4α^+^CK19^+^ cells quickly disappear, underscoring the vital role of continuous chronic injury in driving the transdifferentiation of bile duct cells into hepatocytes (Deng et al., 2018). A recent study in mice lacking FAH and without NTBC treatment revealed severe hepatocyte damage and aging. Under the suppression of the NOTCH signaling pathway, bile duct epithelial cells transdifferentiated into transitional liver progenitor cells (TLPCs) expressing CK19 and HNF4α. This represents a transitional state during the process of BEC transforming into hepatocyte. Activation of the Wnt signaling pathway promoted TLPC differentiation into hepatocytes. These newly formed hepatocytes were capable of division, contributing to LR (Pu et al., 2023). It is worth noting that in addition to BEC being able to transdifferentiate into LPCs, in the case of liver injury induced by DDC, hepatocytes can also give rise to LPCs. LPCs derived from hepatocytes exhibit an upregulation of *mic1-1c3, Sox9, Spp1 (Opn)*, and *Hnf1b* expression, while the expression of *Krt19* and *EpCam* is at moderate levels. The LPCs originating from BEC and those from hepatocytes are not entirely identical. *Sox9, Spp1 (opn)*, and *Hnf1b* show minimal differences between them. Interestingly, LPCs derived from hepatocytes exhibit high expression of *Lgr5*. Compared to hepatocytes, LPCs derived from hepatocytes exhibit downregulation of *Alb, Hgd, Cyp7a1, F9*, and *Hnf4a* expression. Additionally, hepatocyte derived LPC show a noticeable increase in the expression of genes related to the stromal gene (Tarlow et al., 2014). To investigate the mechanism of hepatocyte-derived liver progenitor-like cell generation, Li and others conducted further analysis of the data from Tarlow et al. (2014) They demonstrated that *Arid1a* is essential for hepatocytes to dedifferentiate into liver progenitor-like cells after DDC injury. This group of hepatocytes was confirmed to express *Sox9, Opn*, and *Cd24*. Interestingly, in PV injury models (DDC, 4,4-diaminodiphenylmethane, and bile duct ligation), the loss of Arid1a disrupted the generation of liver progenitor-like cells. However, in CV injury models (TAA and CCl4), the production of liver progenitor-like cells was not observed (Li et al., 2019). However, contradicting evidence suggests that LPCs induced by DDC may not originate from hepatocytes (Malato et al., 2011). In the case of acute CCL4 induced liver injury, regenerating hepatocytes all originate from pre-existing hepatocytes. However, in the context of chronic CCL4 injury, there is evidence of some non-hepatocyte cells contributing to proliferation (Malato et al., 2011). Indeed, it is intriguing that in LR induced by PH, non-hepatocyte-derived cells also participate, but these new hepatocytes are primarily localized around the PV, with limited involvement in other areas (Malato et al., 2011). Telomerase is found in the stem cells of various adult tissues and plays a crucial role in liver diseases. Mutations that activate the TERT promoter are the most frequently occurring mutations in hepatocellular carcinoma (Montgomery et al., 2011; Schepers et al., 2011; Pech et al., 2015). Hepatocytes with high telomerase expression are found in relatively small numbers but are distributed diffusely throughout the entire hepatic lobule. In the homostasis, these cells contribute to hepatocyte production across all lobular zones and undergo self-renewal, giving rise to expanding hepatocyte clones. During injury responses, the regenerative activity of TERT-high hepatocytes accelerates, leading to the generation of hepatocytes dispersed throughout the entire hepatic lobule (Lin et al., 2018). This, to some extent, elucidates the origin of the dispersed proliferating hepatocytes during the regeneration process. While hepatocytes in adult animals do not continuously proliferate (around 84%–90% of hepatocytes undergo division), there is a process of self-renewal (about 10%–15% of hepatocytes divide) that contributes to maintaining liver homeostasis. Evidence suggests that self-renewal in the stable adult liver primarily originates from hepatocytes in the 2 zone. This is mainly attributed to the relatively higher expression levels of Cyclin D1 within this area. Cyclin D1 is a critical protein in the cell cycle, particularly in the G1 phase, and its higher expression likely facilitates cell division in 2 zone hepatocytes to support liver self-renewal (Chen et al., 2020; Wei et al., 2021). In the case of regional liver injury, hepatocytes near the necrotic area primarily proliferate and extend into the damaged region. However, even in hepatocytes further away from the injury site, there is also some compensatory proliferation occurring (Chen et al., 2020; Wei et al., 2021). In cases of repeated and chronic CCl4 induced liver injury, 85% of hepatocytes participate in LR, and they are distributed throughout the hepatic lobule (Chen et al., 2020). The differential expression of Wnt signaling pathway ligands in endothelial cells is an important factor in establishing functional liver lobular zonation. Some researchers have also discovered a group of Axin2+ hepatocytes in the CV area involved in liver self-renewal under the influence of the Wnt signaling pathway. However, this conclusion was soon refuted (Wang et al., 2015; Sun et al., 2020). After labeling cells expressing SOX9 in mouse livers, it was observed that besides high SOX9 expression in bile duct cells, there exists a group of hepatocytes with low SOX9 expression around the PV, referred to as hybrid periportal hepatocytes. It was discovered that this group of hepatocytes exhibits a high proliferative capacity following liver damage (Font-Burgada et al., 2015). In conclusion, with the support of lineage tracing techniques, researchers have gained insights into the origins of regenerating hepatocytes in LR processes induced by various factors. This has allowed for a better understanding of LR from a spatial perspective.

## 6 Time dimension analysis of LR: Regeneration process

PH in rodents is the most commonly used model for studying surgically induced LR. Due to the acute loss of liver parenchymal cells, the remaining hepatocytes quickly re-enter the cell cycle to proliferate, aiming to rapidly restore liver weight and function, thereby meeting the body’s homeostatic requirements. Based on previous research, it is evident that following PH in mice, the residual liver promptly triggers the regenerative process. Within 7 days, the liver essentially regains its original weight (Huang et al., 2022). However, the process of LR is not yet complete at this point. Research suggests that between 7 and 28 days post-surgery, cell aging induced by mechanical stress on endothelial cells is a critical signal for terminating LR (Duan et al., 2022). For the identification of proliferating hepatocytes, commonly used markers include CyclinD1, PH3, Ki-67, PCNA, BrdU/EDU, and others (Langiewicz et al., 2017; Hu et al., 2022; Huang et al., 2022). BrdU and EDU are substances used in cell proliferation studies. When DNA is being replicated, BrdU or EDU can be incorporated into the genomic DNA. This allows researchers to track and identify actively dividing cells by detecting the presence of these analogs in the DNA. Ki67 and PCNA are proteins associated with cell proliferation, and they are continuously expressed throughout the cell cycle. CyclinD1 is a critical protein for cells transitioning from the G1 phase to the S phase of the cell cycle. Interestingly, the regenerative process differs depending on the labeling methods used. Positive cells labeled with markers like PCNA and Ki67 typically emerge in the regenerating mouse liver around 36–40 h post-PH (Huang et al., 2022). In contrast, hepatocytes marked with CyclinD1 exhibit positivity within 12–24 h after the surgery (Hu et al., 2022). Apart from the temporal differences, studies employing Ki67 as a marker for proliferating cells are typically detected at time points around 40–72 h. During this period, the distribution of proliferating hepatocytes appears to be homogenous within the hepatic lobule (Huang et al., 2022). However, in a few studies, a non-uniform distribution of Ki67-positive hepatocytes has also been observed (Chen et al., 2023). However, studies using CyclinD1 as a marker for proliferating hepatocytes often show that CyclinD1 positive hepatocytes are not homogeneously distributed within the hepatic lobule. Instead, they are primarily located in the PV zone and the central region of the lobule at early stage of LR (Hu et al., 2022). Interestingly, a great number of studies have shown that CyclinD1 can also transcriptionally regulate the expression of a large number of genes in addition to regulating the cell cycle (Coqueret, 2002). Overexpression of CyclinD1 in the liver can promote liver hypertrophy, in biological processes associated with CyclinD1 changes, carbohydrates, lipids, amino acids metabolism are among them. Comparing the transcriptional profiles of 70% PH and CyclinD1 overexpression, it was found that there was a highly significant overlap between the two, suggesting that cyclin D1 may regulate a variety of cellular processes in the regenerated liver (Mullany et al., 2008). This effect may also be the reason why the expression of CyclinD1 is inconsistent with the expression of Ki67.

## 7 Cell interaction dimension analysis of LR: Cytokine interaction

### 7.1 Wnt signaling pathway

The Wnt signaling pathway is a group of signal transduction pathways that are activated when Wnt family ligand proteins bind to membrane protein receptors, such as Frizzled (FZD) receptors. This pathway is highly conserved genetically and exhibits significant similarity across various animal species. Mammals have 19 Wnt ligands and 10 FZD receptors, leading to a highly complex cascade of signaling responses (Clevers and Nusse, 2012; Nusse and Clevers, 2017; Jackstadt et al., 2020). The canonical Wnt pathway is the Wnt/β-catenin pathway, where Wnt signaling regulates downstream pathways with β-catenin as a key component. In addition to this, the Wnt pathway can also rely on other molecules to regulate downstream pathways, which are referred to as non-canonical Wnt signaling pathways (Zou and Park, 2023). The regional distribution of Wnt in endothelial cells is considered to be one of the initiating factors for the functional zonation of hepatocytes (Inverso et al., 2021; Zhu et al., 2022). An increasing volume of research indicates that Wnt signaling plays a significant role in hepatocyte proliferation during the process of LR (Inverso et al., 2021; Hu et al., 2022). The conclusions of researches on the canonical Wnt signaling pathway in the process of LR are relatively clear (Yang et al., 2014; Goel et al., 2022). Five minutes after PH, β-catenin levels increase rapidly, followed by a swift nuclear translocation and the expression of target genes (Monga et al., 2001; Torre et al., 2011). Deletion of *Ctnnb1* gene impairs LR in mice subjected to PH. However, this inhibitory effect on LR is only present within the first 48 h (as observed with BrdU-positive hepatocytes) (Sekine et al., 2007). However, another study on mice with *LRP5/LRP6* gene knockout suggests that the inhibitory effect on LR in the absence of these co-receptors lasts for up to 72 h, significantly reducing the rapid proliferative phase of hepatocyte regeneration (Yang et al., 2014). However, more interestingly, a recent study showed that when *Wnt2a* and *Wnt9b* were knocked out in endothelial cells, it not only changed the distribution of functional cell populations in hepatic lobules, but also had a certain inhibitory effect on LR. In mice with *Wnt2a* and *Wnt9b* gene knockout, early-stage CyclinD1 positive hepatocytes after PH are reduced, primarily located in the PV zone. Activation of Wnt receptors can also restore the suppressed proliferation of hepatocytes in the PV zone (Hu et al., 2022). It is worth noting that another factor influencing hepatocyte functional zonation is the differential expression of β-catenin. In the PV zone, the expression of gene adenomatous polyposis coli (APC) leads to the inability of β-catenin to accumulate in the cytoplasm, thereby inhibiting its nuclear accumulation, which affects gene expression (Benhamouche et al., 2006). E-cadherin/β-catenin complex plays an important role in maintaining epithelial junctions. Destruction of this complex affects cell adhesion (Tian et al., 2011). Research has found that during LR following PH, a phenomenon similar to epithelial-mesenchymal transition (EMT) occurs. This involves a decrease in the expression levels of E-cadherin and an increase in the expression levels of mesenchymal genes such as vimentin (Oh et al., 2018). During the initial phases of LR, there is an upregulation of β-catenin expression. Concurrently, the authors have noted an association between E-cadherin and β-catenin, implying that the reduction in E-cadherin and the elevation of β-catenin may complement each other, primarily in the maintenance of intercellular adhesion (Monga et al., 2001). It should be noted that higher levels of E-cadherin expression can buffer the molecular effects of β-catenin, mainly due to the formation of E-cadherin-β-catenin complexes (Huels et al., 2015). Hence, the reduction in E-cadherin expression levels might be one of the ways by which β-catenin dissociates from E-cadherin and gets released, subsequently entering the cell nucleus. In conclusion, the canonical Wnt signaling pathway, primarily governed by β-catenin in the liver, is subject to intricate regulation during the process of LR.

### 7.2 Epidermal growth factor receptor signaling pathway

The human epidermal growth factor receptor family mainly consists of four members, which are HER1, HER2, HER3, and HER4 (Wang, 2017). Research has shown that HER1 (EGFR) and HER3 are expressed in the liver of adult mammals, HER2 is expressed at higher levels in fetal liver and gradually diminishes, and HER4 is almost not expressed in the liver (Carver et al., 2002). Upon ligand binding, the HER family can form homodimers or heterodimers to activate downstream signaling pathways that regulate proliferation and growth. Some ligands, such as amphiregulin (AREG), epidermal growth factor (EGF), heparin-binding EGF (HBEGF), betacellulin (BTC), epiregulin (EREG), and transforming growth factor-alpha (TGF-α), have been shown to activate the EGFR pathway (Singh et al., 2016). The activation of the EGFR signaling pathway represents a robust mitogenic signal in LR. Currently, the ligands that have been extensively studied in the context of LR primarily include EGF, HB-EGF, AREG, EREG, and TGFα (Michalopoulos and Bhushan, 2021). The literature indicates that EGF functions as the PV blood flows from outside the liver into the liver (Olsen et al., 1985; Skov Olsen et al., 1988). Following PH, the transcriptional expression of HBEGF rapidly increases at 1.5 h, peaks at 6 h, and continues to rise until 72 h. After its expression, HBEGF is cleaved by the matrix metalloproteinase family, subsequently released, and exerts both autocrine and paracrine functions (Kiso et al., 1995; Lucchesi et al., 2004; Dong et al., 2020). It is worth noting that the expression of HBEGF primarily occurs in non-parenchymal cells such as Kupffer cells and LSEC, rather than in hepatic parenchymal cells (Kiso et al., 1995). While overexpression of HBEGF does not affect liver size under basal conditions, it accelerates the process of LR after PH (Kiso et al., 2003). AREG and EREG are also crucial ligands for EGFR and play a key role in hepatocyte proliferation (Komurasaki et al., 2002; Liu et al., 2012; Tomita et al., 2014). In rats, the expression levels of AREG increase rapidly after PH and promote hepatocyte proliferation and DNA synthesis through the ERK1/2 and AKT signaling pathways (Berasain et al., 2005). In a liver transplantation model, the induction of AREG expression significantly increased in 50% liver volume transplantation but did not show a significant increase in 30% liver volume transplantation, leading to impaired LR (Liu et al., 2012). Although there was more mechanical stimulation in the 30% liver transplantation model, there was no significant increase in AREG expression. These findings from the literature suggest that the regulation of AREG expression by mechanical stimulation is complex. In addition, cholestatic liver injuries such as primary biliary cirrhosis and primary sclerosing cholangitis can also lead to an increase in AREG expression to protect hepatocytes from injury (Santamaría et al., 2019). Although EREG expression rapidly increases after PH, abnormal EREG expression does not affect the process of LR (Lee et al., 2004). However, EREG can promote the emergence and proliferation of LPC (Tomita et al., 2014). HBEGF and AREG share a common feature, which is that they need to be cleaved by the matrix metalloproteinase family after membrane expression to be released (Lee et al., 2003). Although TGFα expression increases rapidly after PH and is cleaved and released by ADAM17 to activate EGFR, its abnormal expression *in vivo* does not affect LR, similar to EREG (Webber et al., 1993; Russell et al., 1996; Lee et al., 2003). These results suggest that while EGFR activation plays a significant role in LR, the absence of specific EGFR ligands does not necessarily impair this process. The redundancy of multiple ligands provides crucial assurance for EGFR in the LR process. Furthermore, the absence of EGFR only affects LR in the early stages and for a short period of time (Scheving et al., 2015). All those evidences suggests that complex signaling networks are a crucial foundation for the success of LR.

### 7.3 Fibroblast growth factor signaling pathway

In humans, the fibroblast growth factor (FGF) family consists of 22 members, and they initiate signal transduction through the activation of five transmembrane tyrosine kinase receptors, namely, FGFR1-FGFR4 and FGFR5/FGFRL1. This activation mediates various biological effects and plays crucial roles in tumor initiation, development, tissue repair (Maddaluno et al., 2017). In rats, 70% PH increases FGF2 expression and secretion, while a 50% PH does not significantly boost FGF2 secretion (Bönninghoff et al., 2012). Following DDC induced liver injury, there is an increase in FGF7 expression and secretion, which further promotes LPC formation. Mice lacking FGF7 exhibit significantly reduced LPC expansion and higher mortality rates after liver injury (Takase et al., 2013). HSCs are one of the sources of FGF7 during LR (Tsai and Wang, 2011). Most FGFs can transmit signals through interactions with heparan sulfate proteoglycans, however members of the FGF19 subfamily (FGF19, FGF21, and FGF23) show lower affinity for this binding (Goetz et al., 2007; Ornitz and Itoh, 2015). FGF19, FGF21, and FGF23 are increasingly recognized as important regulators of metabolism (Dolegowska et al., 2019). The expression of FGF19 can be triggered by bile acids through multiple farnesoid X receptor (FXR) binding sites in the FGF19 gene (Katafuchi and Makishima, 2022). In wild-type mice, starvation (Byun et al., 2020) and PH(Yang et al., 2013) induce an increase in FGF21 expression levels, and FGF21 not only promotes autophagy, but also increases FFA metabolism in the liver, which can provide energy for LR. In the APAP-induced acute liver injury model, the secretion of FGF21 also rapidly increases (Ye et al., 2014), indicating that liver injury is an important inducer of increased FGF21 secretion. In mice with a hepatocyte-specific knockout of ATG7, the expression level of hepatic FGF21 was significantly increased. This upregulation could partially compensate for the inhibitory effect of reduced autophagy (Kim et al., 2022). Exogenous administration of FGF21 can increase LR and improve survival outcomes following hepatic injury. These effects are primarily executed by inhibiting cell apoptosis and reducing oxidative stress (Qiang et al., 2021). Similarly, in a liver injury model induced by bile duct ligation, injection of FGF19 can also reduce the area of hepatocyte necrosis (Modica et al., 2012). Interfering with FGFRs also has a certain impact on the process of LR. Although some studies suggest that FGFR4 is the only FGF receptor expressed in hepatocytes, while the other FGF receptors are expressed in non-epithelial hepatic cells. However, some studies have found that the knockout of FGFR1 and FGFR2 has a significant impact on the detoxification function of hepatocytes. Still, the peak effect on hepatocyte proliferation during LR is relatively small. In mice with a dominant-negative FGFR2 mutant, LR after a 2/3 PH is severely inhibited. This is mainly because this mutant inhibits signal transduction through different FGF receptors to varying degrees. Although systemic knockout of FGFR4 has little impact on LR, the specific knockout of FGFR4 in mice lacking FGFR1 and FGFR2 significantly inhibits LR. This suggests that to some extent, different FGFRs have functional complementarity, similar to how the single knockout of AKT1 or AKT2 does not inhibit LR (Padrissa-Altés et al., 2015; Valanejad and Timchenko, 2016).

### 7.4 Hepatocyte growth factor signaling pathway

In the intrinsic cells of the LR microenvironment, the main source of hepatocyte growth factor (HGF) is secretion by non-parenchymal cells such as Kupffer cells, HSC, and LSEC (Takeishi et al., 1999; Asai et al., 2006; Lorenz et al., 2018). Neutrophils in the bloodstream can also be induced to express HGF after liver resection surgery, and this may be another important driving factor in promoting LR and repair (Brandel et al., 2022). Evidence suggests that the dominant signaling pathways primarily involved in LR are mediated by EGFR and C-MET (Paranjpe et al., 2016). Hepatocyte Growth Factor (HGF) initially exists in the Pre-proHGF form, which transforms into Pro-HGF when cleaved between Arg494 and Val495. The activation of Pro-HGF necessitates the involvement of various activators, including serine proteases and membrane proteases, such as HGF activator (HGF-A), urokinase-type plasminogen activator (uPA), plasma kallikrein, coagulation factors XII and XI, and metalloproteinases (Zhao et al., 2022). HGF-A is the primary protease in the serum responsible for activating pro-HGF (Fajardo-Puerta et al., 2016). However, interestingly, the loss of HGF-A expression does not appear to have a significant impact on LR, while uPA plays a crucial role in the process of LR (Roselli et al., 1998; Fukushima et al., 2018). HGF expression is rapidly induced following liver resection and CCL4 induced liver injury (Lindroos et al., 1991). Additionally, the exogenous supplementation of HGF can promote LR after injury (Kaibori et al., 2002). Following PH, c-Met tyrosine phosphorylation occurs within 5 min and gradually increases, reaching its peak around 60 min (Stolz et al., 1999). HGF is the exclusive ligand for c-MET, which is a distinct difference from other ligand-receptor interactions, and there are no functionally equivalent substitute factors (Zhao et al., 2022). Once c-MET binds with HGF, it can mediate various downstream signaling molecules such as the MAPK family, JAK/STAT3, and PI3K/AKT, interacting with other ligand-receptor downstream pathways (Zhao et al., 2022). Crosstalk at the cellular level might be an essential mechanism by which the body complements the HGF/C-MET signal.

### 7.5 Vascular endothelial growth factor signaling pathway

During LR, various signaling pathways, including vascular endothelial growth factor (VEGF) -VEGFR, are involved in vascular neogenesis and remodeling (Ross et al., 2001). VEGF is one of the most important members in this context (Bockhorn et al., 2007). VEGF increases after partial liver resection and liver injury, reaching a peak at 72 h, and then gradually decreases (Shimizu et al., 2005). Research has also shown that the levels of VEGF in the PV blood are significantly higher than in the systemic circulation after PH. This is likely because the expression of VEGF increases in the spleen and small intestine during the regenerative process, as the transcription levels of VEGF are elevated compared to the baseline state (Yamamoto et al., 2010). By enhancing the efficiency of its function through combining the collagen-binding domain with native VEGF, researchers have demonstrated that it can promote the process of LR and vascular remodeling (Wei et al., 2022). As previously mentioned, biliary endothelial cells also play a role in LR following severe liver injury. Inhibiting VEGF/VEGFR2 hinders LR mediated by biliary endothelial cells. This effect is primarily exerted through the activation of HSCs, which secrete VEGFA. This further regulates the dedifferentiation process of biliary endothelial cells via the VEGFR2-PI3K-mTORC1 pathway (Cai et al., 2023). Disruptions in the VEGF signaling pathway impact the process of LR. Therefore, the interplay of VEGF signaling between cells is also crucial in the LR process.

### 7.6 Interleukin signaling pathway

The interleukin family is a large group of inflammatory factors, and during the LR process, these inflammatory factors play a crucial initiating role (Michalopoulos and Bhushan, 2021). Among the members of the interleukin family, research on their impact on LR has primarily focused on interleukin-1 (IL-1), IL-2, IL-6, IL-18, IL-22, and IL-33. *In vitro* studies, it has been found that IL-1α does not have a proliferative effect on hepatocytes, while IL-1β can stimulate hepatocyte proliferation and DNA synthesis (Kimura et al., 2014). Research indicates that after PH, the spleen becomes a significant source of rapidly increasing IL-2. The production of IL-2 reaches its peak within 48 h post-PH. Interestingly, despite the increase in IL-2 after PH, the external supplementation of IL-2 significantly inhibits LR. The use of CsA and FK506 to inhibit IL-2 production promotes the process of LR (Tanaka et al., 1993; Wadamori et al., 1996). IL-6 is one of the most extensively studied factors affecting LR. IL-6 drives acute-phase responses, initiates cellular protection, and hepatocyte proliferation through the interaction of IL-6 with IL-6R and the activation of the common receptor glycoprotein 130 (gp130). This activation, in turn, triggers Janus kinases (JAK), signal transducers and activators of transcription (STAT), as well as the PI3K/AKT signaling pathways (Fazel Modares et al., 2019). The functional effects of IL-6 are bidirectional and are contingent on the timing and dosage of the molecule. IL-6-dependent growth-inhibitory effects may result from the increased expression of p21 (Kiseleva et al., 2021). IL-11 is a member of the IL-6 family and, like IL-6, it can bind to IL-6R and GP130 to initiate signal transduction. However, IL-11 has a highly detrimental effect on hepatocytes. A recent study indicated that the IL6:IL6ST fusion protein promotes the repair of APAP-induced liver damage by competitively inhibiting the action of IL-11 (Dong et al., 2021). Similar to IL-2, IL-18 also exhibits inhibitory effects on LR after PH. Although intestinal interleukin-18 (IL-18) production increases significantly within 1 h post-PH, consistent with increased transcription in the intestine, the levels of IL-18 in PV blood peak 24 h after surgery. However, the expression of IL-18 in the liver increases significantly 3 days post-surgery. Deletion of IL-18 and supplementation with recombinant IL-18 binding protein promotes LR (Ma et al., 2020). After PH, the expression levels of IL-22 in the serum increase. Studies have shown that IL-22 promotes the proliferation of hepatocytes *in vitro* and facilitates LR *in vivo* (Ren et al., 2010; Zhang et al., 2016). It is interesting that a recent study suggests IL-22 plays a crucial role in regulating LR and serves as an important link between the brain and the liver. PH results in a significant release of IL-33 into the bloodstream (Nagaoka et al., 2021). IL-33 primarily acts through the ST2 receptor, and in mice lacking IL-33 and ST2 expression, LR is notably inhibited after PH. In more detail, IL-33 acts on enterochromaffin cells, leading to increased serotonin secretion. This, in turn, activates HTR2A/p70S6K in hepatocytes, thus promoting LR (Wen et al., 2023).

### 7.7 Tumor necrosis factor signaling pathway

Tumor necrosis factor-alpha (TNF-α) is a 26-kDa transmembrane precursor protein (mTNFα) that is cleaved by TNF-alpha-converting enzyme (TACE, ADAM17) to generate soluble protein (sTNFα) (Schwarz et al., 2013). Both TNFR1 and TNFR2 receptors can be activated by TNF-α, but they have different distributions. TNFR1 is widely expressed in various tissue cells, while TNFR2 is primarily expressed in endothelial cells. Previous research has shown that TNF-α has a dual role in LR. In routine partial liver resection, the release of TNF-α promotes LR. However, in situations where there is excessive TNF-α release, such as during extensive liver resection, it can have adverse effects on the process of LR. Moderating the production of TNF-α can improve LR in these circumstances (Martino et al., 2012). The above information tells us that interference with TNFα signaling needs to be carried out at three levels, expression, splicing, and receptor binding/downstream signaling factor transduction. Therefore, different scholars have studied the effect of TNF-α on LR from the above different perspectives. Firstly, after partial liver resection, there is an increase in the expression of TACE (Lin et al., 2008). TIMP3 (tissue inhibitor of metalloproteinases 3) can inhibit the activity of TACE, leading to a disruption in TNF-α release. In mice with a deficiency in TIMP3 expression, TNF-α release becomes uncontrolled, accelerating hepatocyte entry into the cell cycle. However, within 72 h, there is significant hepatocytes necrosis. Due to the fact that neutralizing antibodies against TNF-α can rescue hepatocytes necrosis, the authors believe that it is the prolonged action of excessive TNF-α that leads to hepatocytes death (Mohammed et al., 2004). However, it is essential to note that the targets of TACE extend beyond TNF-alpha and also affect EGFR ligands like HBEGF and AREG. Further studies involving mice lacking TNF-alpha expression revealed that these mice did not exhibit significant suppression of LR (48–60 h) and there was no impairment in interleukin-6 release (Fujita et al., 2001; Hayashi et al., 2005). Research on mice lacking TNFR1 expression revealed that these mice experienced impaired LR following PH. This impairment was associated with the suppression of IL-6 expression (Yamada et al., 1997). Similarly, the absence of TNFR1 was found to result in impaired LR in the context of CCL4 induced liver injury (Yamada and Fausto, 1998). Continuous glutathione deficiency hinders the activation of NF-κB, which in turn disrupts the process of LR in response to TNFα/TNFR1 signaling (Riehle et al., 2013). The results from the above studies indicate that the presence of TNFR1 and the successful activation of NF-κB may be critical factors affecting LR, rather than the TNFα itself.

### 7.8 Hedgehog (HH) signaling pathway

Hedgehog (HH) is a crucial signaling pathway that plays a pivotal role in regulating various cellular processes, including proliferation, apoptosis, migration, and differentiation. The Hh signaling pathway is initiated when three ligands, namely, SHH, IHH, and DHH, bind to receptors. Its primary effects are mediated through three transcription factors from the GLI family, which includes GLI1, GLI2, and GLI3 (Omenetti et al., 2011; Gao et al., 2018). Following a 70% PH, there is a substantial increase in the expression of Shh and Ihh in the liver, which actively participate in the process of LR (Ochoa et al., 2010). In another study, in order to find the difference in the mechanism of promoting LR between ALPPS and PVL and PH, the researchers analyzed the transcriptome data during the regeneration process and further verified it. It was found that ALPPS surgery caused an increase in IHH secreted by activated HSCs, and activated the HH signaling pathway in hepatocytes to promote the accumulation of GLI1 in the nucleus, thereby promoting LR (Langiewicz et al., 2017; 2018). Disturbing the transmission of HH signaling pathway will interfere with the process of LR. Vismodegib, a smo antagonist, significantly inhibited the process of LR (Tao et al., 2022). In addition to the mechanism of the HH signaling pathway itself to promote regeneration, HH signaling can also regulate the process of LR through Hippo/Yap signaling (Swiderska-Syn et al., 2016).

### 7.9 Transforming growth Factor-β1 signaling pathway

There are 33 members of the transforming growth factor-β (TGF-β) family in mammals, and TGF-β protein includes three subtypes (TGF-β1, TGF-β2 and TGF-β3) (Derynck and Budi, 2019). In general, TGF-β1 is the most widely and deeply studied subtype of liver fibrosis (Dewidar et al., 2019). Although TGF-β1 is a clear and stable mitogenic inhibitor of hepatocyte proliferation, it also plays an important role in the progression of liver cancer (Fausto et al., 1991; Zaidi et al., 2022). One hour after PH, TGF-β1 was released from the liver to reduce its content. This change may play an important role in initiating LR. The change of hepatocyte proliferation is gradually carried out from the PV to the central vein with the release of TGF-β1 in hepatocytes (Jirtle et al., 1991). However, the expression of TGF-β1 also changed during LR. The expression of TGF-β1 mRNA increased at 3 h after PH and reached a plateau at 72 h (Jakowlew et al., 1991). TGFβ1 levels rise during LR but do not hinder the process. How do cells evade TGF-β1’s inhibitory effects? Studies have shown that the expression level of TGF-β1 receptor in the liver is reduced during regeneration (Chari et al., 1995). This weakened the inhibitory effect of TGF-β1 on hepatocyte proliferation to a certain extent. A study has shown that the activation of TGF-β1 signaling pathway and EMT coexist in the early stage of LR after PH (Oh et al., 2018). Loss of YAP1 expression significantly inhibits the activation of the TGF-β1 signaling pathway, resulting in the reversal of EMT and reduced cell proliferation (Oh et al., 2018). Compared to normal hepatocytes, HCC cells exhibit a significant EMT, with suppressed epithelial characteristics and increased expression of mesenchymal genes (Giannelli et al., 2016). EMT may be the partial reason why two different types of cells respond differently to TGF-β1. The mechanisms underlying EMT during LR are not fully understood, and the significance of EMT in the LR process remains to be fully clarified, requiring further research.

### 7.10 Other signaling pathway and extracellular vesicle effects

As mentioned earlier, LR is a process involving the interaction of various signaling molecules between cells. Therefore, beyond the 9 families we discussed previously, there are still many signaling molecules that mediate interactions between cells in the process of LR. Platelet-derived growth factor was initially discovered as a mitogen found in platelets, capable of promoting cell division (Ross et al., 1990; Qian et al., 2020). Insulin is primarily a hormone secreted by the pancreatic beta cells, and its main function is to regulate the stability of blood glucose levels in the body. However, studies indicate that in rats with insulin expression deficiency, posthepatectomy LR is significantly inhibited (Tseng et al., 2011). Leptin, secreted by adipocytes, regulates the body’s lipid metabolism. By binding to specific receptors, it can additionally stimulate cell proliferation in the regenerating liver (Cilekar et al., 2013). While LR after partial hepatectomy is inhibited in mice lacking glucocorticoid receptors, the exogenous supplementation of glucocorticoids suppresses DNA synthesis during LR process (Tsukamoto and Kojo, 1989; Shteyer et al., 2004). Interferon alpha is also involved in the LR process mediated by oval cells after liver injury (Batusic et al., 2011). In addition to protein molecules, sphingosine-1-phosphate, prostaglandin E2, and serotonin are also involved in the regulation of LR (Ding et al., 2016; He et al., 2019; Cheng et al., 2021). Extracellular vesicles represent another form of mediating communication between cells (Brandel et al., 2022), and due to their accessibility, extracellular vesicles have become an important approach for the diagnosis and treatment of various diseases (Sun et al., 2023). After liver resection, extracellular vesicles produced by hepatocytes can stimulate the production of neutrophils and release cytokines such as HGF, promoting LR (Brandel et al., 2022). Extracellular vesicles derived from mesenchymal liver cells can effectively ameliorate aging and promote LR (Zhang et al., 2023). With the growing appreciation of the pivotal role played by extracellular vesicles, the synergy between nanomaterial technology and extracellular vesicles is emerging as a leading trend in the field of regenerative medicine (Li et al., 2022).

## 8 Evolution and potential mechanisms of LR pattern

**FIGURE 4:**
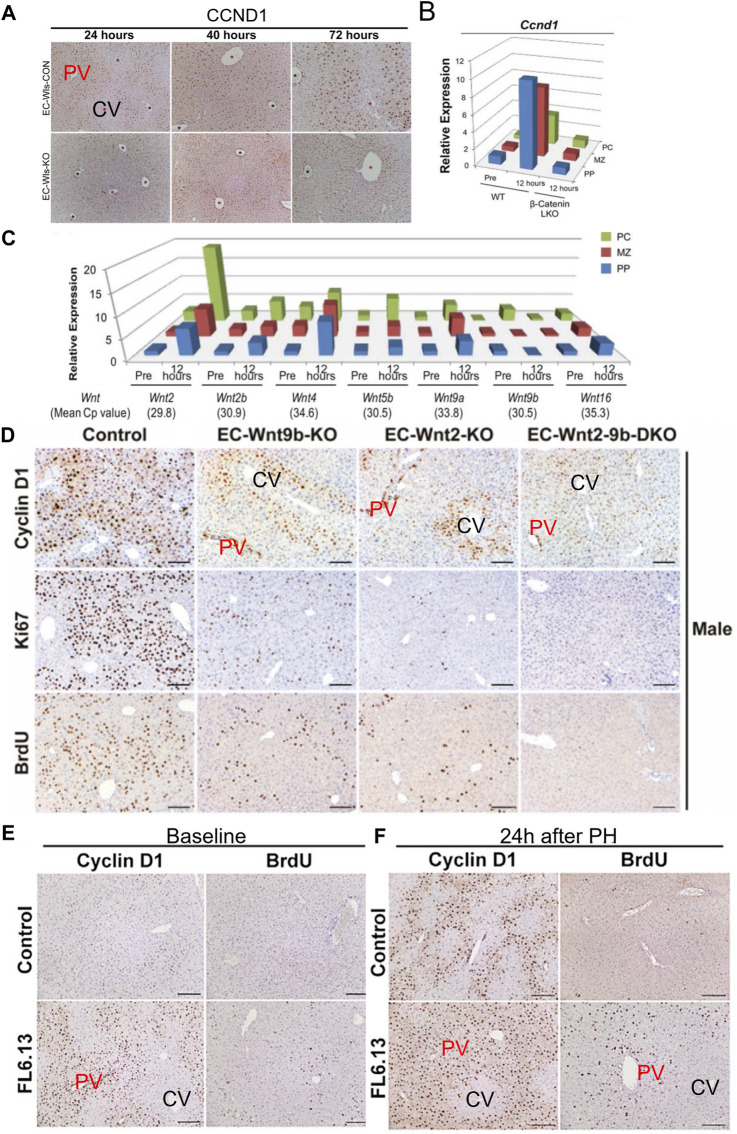
Potential Evidence I of Liver Regeneration Dynamics. **(A–C)** Expression of CyclinD1 in hepatocytes and Wnt family genes expression from different regions following partial hepatectomy, adapted with permission from Preziosi et al. (2018). Copyright © 2018 The Authors. **(D–F)** Impact of wnt2a and wnt9b knockout, as well as LRP6 activation, on the proliferation of hepatocytes in the PV area during the regeneration process, Reprinted with permission from Hu et al. (2022), Copyright © 2022 Elsevier.

**FIGURE 5:**
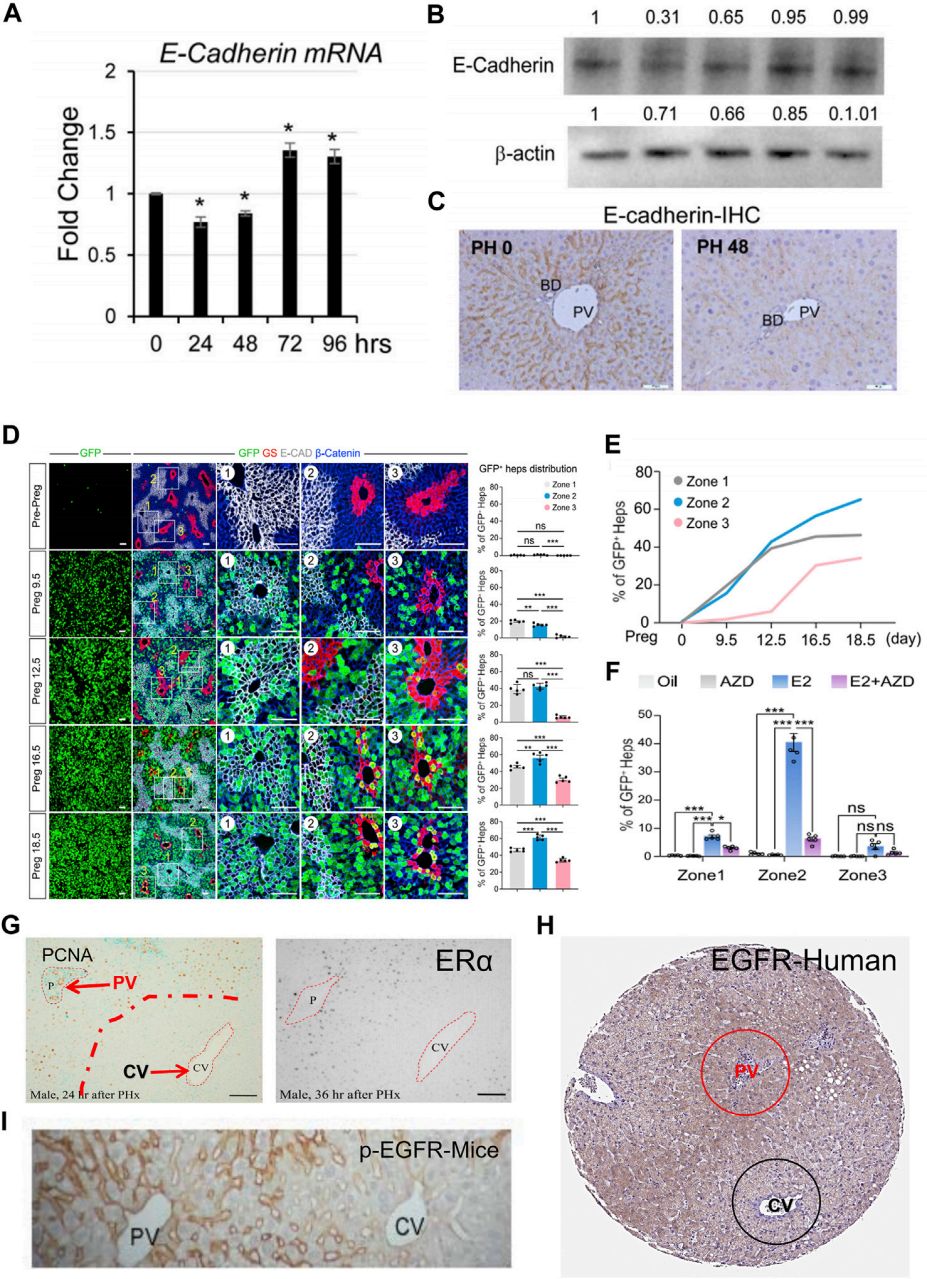
Potential Evidence II of Liver Regeneration Dynamics. **(A–C)**, After partial hepatectomy, the expression level of E-cadherin decreased [Reprinted with permission from Oh et al. (2018), Copyright © 2018 Elsevier.] **(D–F)**, The liver of mice increased after pregnancy, and the proliferation of hepatocytes was more in zone 1 and zone 2 [Reprinted with permission from He et al. (2023), Copyright © 2023 Elsevier.] The difference of proliferating hepatocytes in zone 1 and zone 2 caused by pregnancy was much smaller than that caused by estrogen. **(G)**, The early distribution of estrogen receptor α after partial hepatectomy in male rats was similar to that of PCNA-positive hepatocytes [reprinted with permission from Batmunkh et al. (2017), Copyright 2017 The Japan Society of Histochemistry and Cytochemistry]. **(H)**, Immunohistochemical staining of EGFR in human specimens in HPA database (https://www.proteinatlas.org). **(I)**, PV infusion of EGF resulted in increased p-EGFR expression in PV area hepatocytes than in CV area [Reprinted with permission from Scheving et al. (2015), Copyright © 2015 the American Physiological Society].

Regional liver injuries, such as those induced by CCL4 or APAP, lead to hepatocyte necrosis in the CV area. Since there is no loss of liver lobes, the liver typically regains its original structure and function within a week. In the case of injuries caused by APAP and CCL4, the primary effect is hepatocyte necrosis, with minimal damage to non-parenchymal cells and extracellular matrix components. As previously described, proliferating hepatocytes are mainly distributed in non-injured areas. Hepatocytes gradually migrate from the surrounding necrotic area into the necrotic zone until the original liver lobular structure is restored. Endothelial cells in the CV area provide a highly Wnt signaling environment that further transform the newly formed hepatocytes, allowing to regain full liver lobule functionality. Non-regional liver injuries are typically studied in zebrafish or rodent models to simulate the process of LR observed clinically after PH. With advancements in clinical surgical techniques, animal models have evolved to include PVL, PVE, and ALPPS. These models aim to more closely replicate clinical scenarios for the study of LR. However, in the case of non-regional liver injuries, hepatocytes may or may not experience acute loss, and there is no damage to the cells or structure within the liver lobules where regeneration occurs. In 1985, a researcher proposed that hepatocytes are generated around the PV area and then gradually migrate towards the CV area (Zajicek et al., 1985). The findings of hepatocyte lineage research in 1994 contradicted the theory of streaming liver (Bralet et al., 1994). However, in 2006, researchers proposed that after PH, proliferating hepatocytes gradually spread from the PV area towards the CV area, accompanied by phenomena such as cell apoptosis in the CV area (Baier et al., 2006). Currently, the concept of the “streaming liver” model is rarely mentioned, but the non-uniform distribution of proliferating hepatocytes during LR persists in several research findings (Baratta et al., 1996; Baier et al., 2006; Batmunkh et al., 2017; Hu et al., 2022; Chen et al., 2023). This non-uniform distribution (Regeneration Pattern evolution) primarily manifests as the gradual dispersion of proliferating hepatocytes from the PV area towards the CV area, ultimately diffusing throughout the entire liver lobule. Our results have confirmed the accuracy of this regeneration pattern in a variety of surgical models in rats and mice. However, the mechanism of the evolution of LR is not clear, and different studies have infiltrated different information. These results suggest the complexity of this phenomenon. The study of Hu S et al. reflected the phenomenon of regional distribution of proliferating hepatocytes during regeneration (Figure 4) (Hu et al., 2022). In *Wnt2a* and *wnt9b* knockout (KO) mice, the process of male and female regeneration showed significant differences, which may be related to the difference in the basic expression of estrogen receptor alpha (ERα). In male mice, *wnt2a*-KO, *wnt9b*-KO and *wnt2a-wnt9b*-DKO groups showed significant inhibition of hepatocyte proliferation in the PV region. The expression of *wnt2a* and *wnt9b* in the basic state of the liver was mainly in the CV area of LSEC, but in the process of LR, the expression of *wnt2a* increased in the three regions, mainly in the CV area (Preziosi et al., 2018). The main effector transcription factor of Wnt signaling pathway is β-catenin. The author speculates that the reason for the late proliferation of this CV region may be related to the occupation of β-catenin and genes such as Cyp2e1 and Gls in the basic state (Preziosi et al., 2018). While the zonation of Wnt expression in endothelial cells is considered to be the basis for the functional zonation of the liver, the differential distribution of β-catenin in hepatocytes is related to the expression differences of APC, as APC primarily mediates the degradation of β-catenin (Benhamouche et al., 2006). In addition to the role of transcription factors, β-catenin also has the function of cell connection. This function is mainly carried out by forming a complex with E-cadherin (Tian et al., 2011). E-cadherin in the liver is mainly distributed around the PV region and exists as a marker gene for cells in the PV region. Studies have shown that there is a significant decrease in the expression level of E-cadherin during LR (Figures 5A–C) (Oh et al., 2018). The downregulation of E-cadherin expression may be a way to increase the release of β-catenin and further enter the nucleus, which may also be one of the reasons for promoting the proliferation of hepatocytes from the PV region to the CV region. Batmunkh et al. discovered that ERα is involved in regulating LR, with significant differences in its expression between males and females. In the early stage of regeneration after liver resection in male rats, the increased expression of ERα in the PV area is consistent with the distribution of proliferative hepatocytes, which may promote the diffusion of proliferative hepatocytes from the PV area to the CV area (Figure 5G) (Batmunkh et al., 2017). In a recent study on the mechanism of liver enlargement in mice during pregnancy, ERα in the liver of female mice did not show regional distribution in hepatic lobules. Estrogen treatment can increase liver size by relying on CyclinD1 expression. It can be seen from the source of cell proliferation that all hepatocytes proliferate, but the hepatocytes in zone 2 are far more than the hepatocytes in zone 1 and zone 3. Inhibition of estrogen receptor α does block the proliferation of hepatocytes in all zones. Although the estrogen/estrogen receptor α/CyclinD1 axis explains the phenomenon of hepatocyte proliferation in zone 2, it still fails to fully explain the mechanism of liver enlargement during pregnancy. This is mainly due to the fact that there is no significant difference in hepatocyte proliferation between zone 1 and zone 2 during pregnancy for a long period of time, but in estrogen-induced hepatocyte proliferation, zone 2 hepatocytes showed much higher proliferation than zone 1 hepatocytes (Figures 5D–F) (He et al., 2023). Therefore, the proliferation of zone 1 hepatocytes induced by pregnancy may be partly due to the estrogen effect, and the deeper mechanism still needs to be further explored. EGFR is involved in the process of LR. From the results of the public database, the distribution of EGFR protein expression is higher in the PV area than in the CV area. Studies have found that after PV injection of EGF, the distribution of p-EGFR in the liver of mice is also higher in the PV area than in the CV area. EGF mainly comes from the synthesis of the duodenum and flows into the liver through the PV. This physiological distribution of EGFR and the direction of blood flow may determine the gradual diffusion of proliferating hepatocytes from the PV region to the CV region (Figures 5H, I) (Scheving et al., 2015). After PH, TGF-β1 is released from the liver to reduce the content in the liver. This change may play an important role in initiating LR. The change of hepatocyte proliferation is gradually carried out from the PV region to the CV region with the release of TGF-β1 in hepatocytes (Jirtle et al., 1991). In short, the process and molecular mechanism of LR are complex, which may involve the spatial distribution of gene expression in hepatocytes, endothelial cells and stellate cells, and also with the assisting of the physiological blood flow direction of hepatic lobules. This is a process that needs further exploration.

## 9 Perspective

To thoroughly analyze the process of LR, it is crucial to fully understand the pattern of LR. The process of LR needs to be examined from three dimensions. First, the spatial dimension: The liver lobule serves as the fundamental structural and functional unit of the liver, with its unique spatial structure providing a critical foundation for the distinctiveness of LR. Different regions of the liver contain hepatocytes with distinct functions, indicating that hepatocytes in different regions may play various roles and exhibit varying advantages during the process of LR. Second, the temporal dimension: LR can be divided into initiation, regeneration, and termination stages. Nevertheless, as time progresses, it is essential to understand how regenerating hepatocytes evolve and distribute within the liver lobules. Third, the dimension of cell interactions: This dimension includes two critical aspects. First, it involves an examination of how liver cells communicate with cellular components in the regenerative microenvironment through signaling molecules, which are essential for maintaining the process of LR. Second, it is vital to consider how the distribution of molecular signals changes as time progresses. A clear understanding of these three dimensions is pivotal for recognizing and comprehending the patterns of LR, and it is crucial for effectively controlling LR.
